# Recent Progress on the Molecular Mechanism of Quality Controls Induced by Ribosome Stalling

**DOI:** 10.3389/fgene.2018.00743

**Published:** 2019-01-17

**Authors:** Ken Ikeuchi, Toshiaki Izawa, Toshifumi Inada

**Affiliations:** Gene Regulation Laboratory, Graduate School of Pharmaceutical Sciences, Tohoku University, Sendai, Japan

**Keywords:** ribosome, ribosome stalling, no-go mRNA decay, ribosome-associated quality control, ribosome ubiquitination

## Abstract

Accurate gene expression is a prerequisite for all cellular processes. Cells actively promote correct protein folding, which prevents the accumulation of abnormal and non-functional proteins. Translation elongation is the fundamental step in gene expression to ensure cellular functions, and abnormal translation arrest is recognized and removed by the quality controls. Recent studies demonstrated that ribosome plays crucial roles as a hub for gene regulation and quality controls. Ribosome-interacting factors are critical for the quality control mechanisms responding to abnormal translation arrest by targeting its products for degradation. Aberrant mRNAs are produced by errors in mRNA maturation steps and cause aberrant translation and are eliminated by the quality control system. In this review, we focus on recent progress on two quality controls, Ribosome-associated Quality Control (RQC) and No-Go Decay (NGD), for abnormal translational elongation. These quality controls recognize aberrant ribosome stalling and induce rapid degradation of aberrant polypeptides and mRNAs thereby maintaining protein homeostasis and preventing the protein aggregation.

## Introduction

Protein synthesis is a fundamental step of gene expression in all organisms. Translation elongation is perturbed by unique sequences, for instance poly-adenosine tract (Dimitrova et al., 2009), tandem rare codons such as yeast arginine CGA rare codon (Doma and Parker, 2006; Chen et al., 2010; Letzring et al., 2013), inhibitory di-codon pairs (Gamble et al., 2016), oxidized RNA (Simms et al., 2014) and robust higher order mRNA structures (Doma and Parker, 2006; Tsuboi et al., 2012), or by the severe cellular conditions including amino acid starvation (Guydosh and Green, 2014), tRNA deficiency (Ishimura et al., 2014), oxidative stress (Simms et al., 2014) and genetic mutations (LaRiviere et al., 2006). Ribosome stalling on the specific sites result in the perturbation of ribosome recycling as well as the production of aberrant truncated proteins. Cells have quality control systems to recognize ribosome stalling and eliminate the aberrant mRNAs and proteins. The stalled ribosome by the tandem CGA codons or KKK codon cluster is subjected to Ribosome-associated Quality Control (RQC) that induces co-translational degradation of the arrest products (Dimitrova et al., 2009; Bengtson and Joazeiro, 2010; Brandman et al., 2012). RQC machinery is well conserved from yeast to human cells and related not only in cytosolic protein quality control but also in mitochondrial function, protein aggregation, and neurodegeneration (Wilson et al., 2007; Chu et al., 2009; Bengtson and Joazeiro, 2010; Brandman et al., 2012; Brandman and Hegde, 2016; Shao and Hegde, 2016; Garzia et al., 2017; Matsuo et al., 2017; Sitron et al., 2017; Sundaramoorthy et al., 2017; Juszkiewicz et al., 2018; Kuroha et al., 2018; Szadeczky-Kardoss et al., 2018; Verma et al., 2018; Zurita Rendon et al., 2018). No-go mRNA decay (NGD) is a eukaryotic mRNA quality control system and triggered by endonucleolytic cleavage in the vicinity of the stalled site followed by the exoribonucleolytic decay (van Hoof et al., 2002; Doma and Parker, 2006). In this review, we mainly describe the mechanism of RQC and NGD in yeast because it has been extensively investigated in yeast.

## Ribosome-Associated Quality Control

### Ribosome Ubiquitination Is Required for the Subunit Dissociation in RQC

**FIGURE 1:**
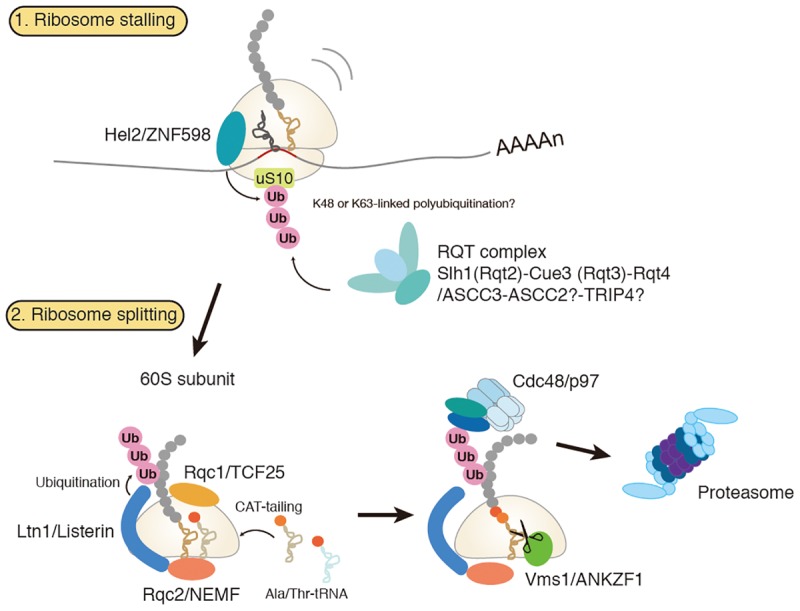
Amodel for ribosome-associated protein quality control (RQC). Hel2/ZNF598 binds to the stalled ribosome and mediates ubiquitination of uS10 protein of 40S ribosomal subunit. Subsequently, RQT complex, which is composed of Slh1/Rqc2, Cue3/Rqt3, and Rqt4, binds to the ubiquitylated ribosome to trigger splitting of ribosome and RQC. Stalled polypeptide on 60S ribosome is ubiquitylated by E3 ligase Ltn1 in concerted action with Rqc1. Rqc2 mediates elongation of stalled polypeptides by the C-terminal addition of multiple alanyl and threonyl residues (CAT-tailing). A ubiquitylated polypeptide is released by Vms1 and extracted by Cdc48 for proteasomal degradation.

Dissociation of stalled ribosomes into 40S and 60S subunits is an essential step to initiate RQC. Recent studies have demonstrated that ubiquitylation of stalled ribosomes triggers the subunit dissociation. An E3 ubiquitin ligase Hel2 in yeast and its mammalian homolog ZNF598 play a crucial role in this process (Brandman et al., 2012; Letzring et al., 2013; Saito et al., 2015; Garzia et al., 2017; Matsuo et al., 2017; Sitron et al., 2017; Sundaramoorthy et al., 2017; Juszkiewicz et al., 2018). A current model of RQC-trigger pathway induced by ribosome stalling is shown in Figure 1. Hel2 was initially identified as a Histone E3 ligase 2 required for cytosolic excess histone proteins (Singh et al., 2012). Hel2 recognizes the ribosomes stalled at the CGA rare codon cluster or poly(A) stretches on the mRNA sequences and mediates K63-linked poly-ubiquitination of ribosomal small subunit uS10 at K6 and K8 (Matsuo et al., 2017; Ikeuchi et al., 2019). The resulting ubiquitinated ribosomes are split into the subunits and then subjected to the downstream RQC pathway (Matsuo et al., 2017). ZNF598 in mammals also recognizes stalled ribosomes at the poly(A) stretches. ZNF598 is located in the head region of the 40S subunit and ubiquitinates both uS10 (at K4 and K8) and eS10 (at K138 and K139) (Garzia et al., 2017; Sundaramoorthy et al., 2017; Juszkiewicz et al., 2018). Asc1 in yeast and its mammalian homolog RACK1 are also required for stall-mediated early RQC pathway (Kuroha et al., 2010; Letzring et al., 2013; Sitron et al., 2017). RACK1 is responsible for ZNF598-mediated ribosome ubiquitination in human cells (Sundaramoorthy et al., 2017; Juszkiewicz et al., 2018), and Asc1/RACK1 locates on the 40S head near the Hel2/ZNF598 target proteins, uS10, eS10, and uS3. Notably, Asc1 is not essential for non-stop RQC (Matsuda et al., 2014; Ikeuchi and Inada, 2016), because Asc1 is vital for stalling on poly(A) sequence but not for Dom34-mediated ribosome splitting at the 3′ end of the non-stop mRNA(Ikeuchi and Inada, 2016).

### A Disome as a Structural Unit for RQC

Recent studies strongly suggest that a di-ribosome (disome) is a structural unit for RQC (Ikeuchi et al., 2019; Juszkiewicz et al., 2018). Hegde and co-workers demonstrated that ZNF598 preferentially ubiquitinates the disome with the mammalian *in vitro* translation system (Juszkiewicz et al., 2018). The collided di-ribosome is a minimal unit for RQC by ZNF598. The collided di-ribosome structure reveals a broad 40S – 40S interface where the ubiquitination target of ZNF 598 is present (Juszkiewicz et al., 2018). It was proposed that the use of ribosomal collisions on behalf of stall makes it possible to adjust the degree of acceptable deceleration by the initiation rate for that mRNA (Juszkiewicz et al., 2018).

In yeast, CGACCG repeat induces RQC and NGD quality controls *in vivo* (Ikeuchi et al., 2019). The disomes formed by the CGACCG repeat are preferred as targets for Hel2-mediated uS10 ubiquitination over monosomes *in vitro* translation system (Ikeuchi et al., 2019). The Cryo-EM structure of the disome revealed that the leading ribosome is stalled in the classical POST-translocation state with an empty A-site and occupied P- and E-sites. The second ribosome is locked in an incomplete translocation step, and in a hybrid state with A/P and P/E-tRNAs. The interface between the leading and the colliding ribosomes is mainly formed by the small 40S subunit (Ikeuchi et al., 2019). The Cryo-EM analysis of Hel2-bound ribosome revealed that Hel2 preferentially binds to the rotated ribosome with the hybrid tRNAs (Matsuo et al., 2017). Therefore, it is possible that Hel2 binds to the colliding ribosome because it is the rotated form with the hybrid tRNAs. Importantly, the 40S interribosomal contact interface brings all proteins targeted by Hel2 during quality control nearby. Moreover, both Asc1 (RACK1 in humans) molecules are in direct contact forming one of the inter-ribosomal interaction sites in a disome. It may represent the ideal substrate for Hel2, thereby specifically recognizing a prolonged translation stall to initiate RQC by its E3 ubiquitin ligase activity.

### The RQT Complex Has Potential to Dissociate the Subunit in RQC

Recent studies have identified the RQC trigger (RQT) complex that is associated with translating ribosomes and essential for dissociation of stalled ribosomes (Matsuo et al., 2017). The RQT complex is composed of double RecA helicase domain-containing protein Slh1/Rqt2, CUE domain-containing ubiquitin-binding protein Cue3/Rqt3 and C2HC5-type zinc-finger (ZnF) protein Ykr023w /Rqt4 (Matsuo et al., 2017; Sitron et al., 2017). The RQT function depends on the ATPase hydrolysis motif of Slh1/Rqt2 and moderately accelerated by the CUE domain of Cue3, indicating that the RQT complex-mediated recognition of the ubiquitin on the ribosome promotes ribosome splitting. It remains elusive how the RQT complex acts to induce splitting of the stalled ribosome and how helicase activity of Slh1 works on the stalled complex. Additionally, the function of Rqt4 and the role of its unique ZnF domain are still undefined. In a mammal, ASCC3 has similarity to yeast Slh1/Rqt2 and is required for mammalian RQC induction (Matsuo et al., 2017). Human orthologs of Rqt3-4 remain to be clarified. It has been reported that ASCC3 forms ASC-1 complex (ASCC) with CUE domain containing protein ASCC2, RNA ligase-like protein ASCC1 and C2HC5-Znf protein TRIP4/ASC-1 (Jung et al., 2002). Moreover, ASCC with demethylase ALKBH3 forms complex to repair DNA alkylation in nuclear foci, and ASCC2 binds to K63-linked ubiquitin via its CUE domain, is critical for ASCC-ALKBH3 recruitment in the repairing foci (Brickner et al., 2017). At the moment, ASCC2 and TRIP4 are the potential mammalian orthologs of Cue3/Rqt3 and Rqt4, respectively, and might act as the mammalian RQT-complex.

### Quality Control of Nascent Proteins on the 60S Subunit

Stalling of ribosome can generate faulty proteins with cytotoxic properties. To prevent accumulation of such toxic proteins, cells have evolved the RQC complex that targets them for proteasomal degradation (Bengtson and Joazeiro, 2010; Brandman et al., 2012; Defenouillere et al., 2013; Verma et al., 2013). RQC complex consists of Ltn1, Rqc1, Rqc2, and Cdc48 (Listerin, TCF25, NEMF and p97 in a mammal, respectively). RQC complex is recruited to the 60S subunit-peptidyl-tRNA complex after splitting of the stalled ribosome (Shao et al., 2013; Shao and Hegde, 2014). Rqc1 is also involved in this ubiquitination step, yet its function is little understood (Brandman et al., 2012; Defenouillere et al., 2013). Rqc2 facilitates recruitment of Ltn1 to the 60S subunit (Lyumkis et al., 2014; Shao et al., 2015). Rqc2 also mediates elongation of stalled nascent polypeptides on the 60S subunit by the C-terminal addition of multiple alanyl and threonyl residues (CAT-tailing) in a template-free and 40S subunit independent manner (Shen et al., 2015). A recent study has shown that CAT-tailing acts as a fail-safe mechanism for efficient ubiquitination by Ltn1, by extracting the lysine residues sequestered in the ribosomal tunnel to the cytosol so that the lysine residues are accessible to Ltn1 (Kostova et al., 2017). Cdc48/p97, together with its co-factors Ufd1 and Npl4, acts in the downstream step to extract the nascent polypeptides for proteasomal degradation (Brandman et al., 2012; Defenouillere et al., 2013; Verma et al., 2013). An important question remaining to be solved is the mechanism for releasing the peptidyl-tRNA from the 60S subunit. Recent studies have identified Vms1 (ANKZF1 in a mammal) as a peptidyl-tRNA hydrolase in RQC. Vms1/ANKZF1 has a eukaryotic release factor 1 (eRF1) like domain with the conserved Gln residue that is supposed to catalyze peptidyl-tRNA hydrolysis in a similar way to the Gln residue of conserved GGQ motif in eRF1 (Verma et al., 2018; Zurita Rendon et al., 2018). However, the GGQ motif is not conserved, but is deviated to GSQ in yeast Vms1 and even to TAQ in human ANKZF1. More recently, ANKZF1 has been reported to function as a tRNA endonuclease rather than as a peptidyl-tRNA hydrolase *in vitro* (Kuroha et al., 2018). Future structural and biochemical studies will uncover the detailed mechanism of peptidyl-tRNA hydrolysis and tRNA cleavage by Vms1/ANKZF1.

Failure of ubiquitination by a loss of Ltn1 function causes accumulation of CAT-tailed proteins (Shen et al., 2015). Recently, several groups have reported that CAT-tailed proteins have a strong propensity to aggregate and cause proteotoxic stress in yeast (Choe et al., 2016; Defenouillere et al., 2016; Yonashiro et al., 2016; Izawa et al., 2017). They sequester multiple essential chaperones and form SDS-resistant aggregates, thereby interfering with general protein quality control pathways. CAT-tailed proteins have also reported to have an extremely toxic effect on mitochondria. The CAT-tailed mitochondrial proteins, synthesized in the cytosol but once imported into the mitochondria, sequester multiple essential chaperones, proteases and the components of translation machinery in the mitochondrial matrix, resulting in defective assembly of respiratory chain complexes and cell death (Izawa et al., 2017). In this context, Vms1 also acts as a key player to protect mitochondria from the toxic effect of CAT-tailed mitochondrial proteins. Vms1 antagonizes Rqc2, thereby preventing CAT-tailing and facilitating the release of stalled mitochondrial proteins to the downstream quality control network in the mitochondrial matrix (Izawa et al., 2017). Vms1 is particularly crucial for stalled mitochondrial proteins that cannot be ubiquitylated by Ltn1 due to the coupling of translation and protein import across the mitochondrial membranes. Since all the RQC components are conserved in eukaryotes including human, clearance of stalled proteins may be of general significance for cellular homeostasis. Indeed, the *listerin* hypomorphic mouse was shown to cause neurodegeneration (Chu et al., 2009). Further studies will clarify the roles of RQC in protein aggregation, mitochondrial dysfunction, and disease progression.

## No-go mRNA Decay and Roles of Quality Control Factors

**FIGURE 2:**
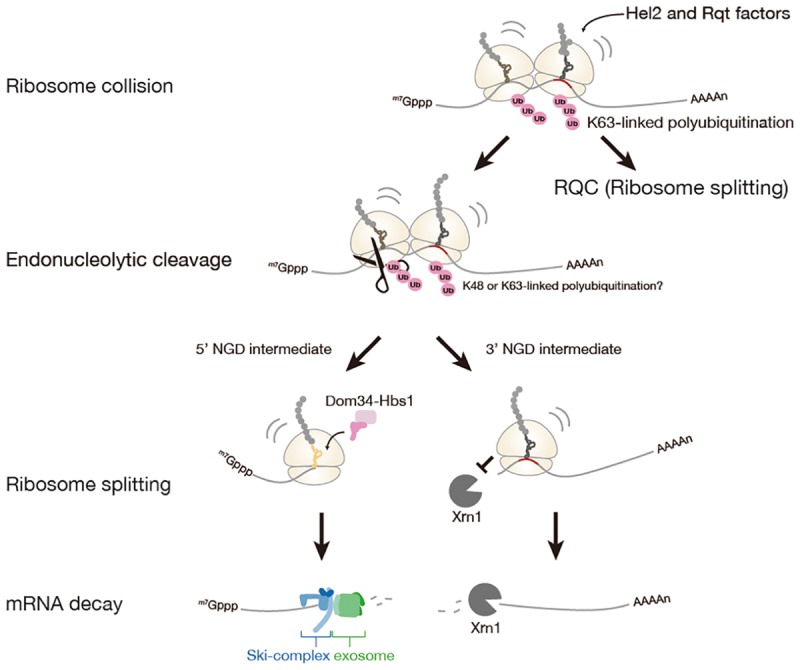
No-Go mRNA decay triggered by ribosome stalling. A collision of stalled ribosomes leads to ubiquitination of 40S ribosomal subunit and endonucleolytic cleavage of mRNA. The stalled ribosome at the 3′-end of 5′-NGD intermediate is split by Dom34: Hbs1 complex and the 5′-NGD intermediate is degraded by Ski complex and exosome. The stalled ribosome on the 3′-NGD intermediate may be dissociated or engaged in a restart of translation, and the 3′-NGD intermediate is degraded by Xrn1.

No-go mRNA decay (NGD) is a cytosolic quality control system for mRNA induced by the ribosome stalling. NGD system is firstly discovered in *Saccharomyces cerevisiae* (Doma and Parker, 2006). NGD is conserved in fruit fly and plant, yet it has not been characterized in a mammal (Passos et al., 2009; Szadeczky-Kardoss et al., 2018). The NGD is triggered by endonucleolytic cleavage of mRNA in the vicinity of the stalled ribosome, then the resulting the 5′- and 3′-fragments are rapidly degraded by the exoribonucleolytic cleavages (Figure 2).

### Roles of Ribosome Collision and Ribosome Ubiquitination in NGD

A recent study has proposed that ribosome collision is a critical trigger for mRNA cleavages in the initial step of NGD (Simms et al., 2017). In the study, accumulation of ribosomes on the mRNA has been observed as multiple cleavage sites which were distributed 43∼300 nt upstream of the stalling site with approximately 30 nt periodicity likely due to the stacked ribosomes array. Similar situations that can be evidence for ribosome collision were observed on the *GFP-Rz* (hammerhead ribozyme) auto-cleaved truncated stop-codon-less reporter mRNA or the endogenous truncated mRNA under the *dom34* deletion condition (Tsuboi et al., 2012; Guydosh and Green, 2014; Ikeuchi and Inada, 2016). Moreover, the collision of ribosome seems to be required for the cleavages of mRNA, as cleavage efficiency of mRNA was decreased with reduced initiation efficiency by using long 5′-UTR. Hel2 was also proposed as a factor involved in NGD, and Hel2-mediated ubiquitination of ribosomal protein uS3 associates with ribosome collision. However, the requirement of Hel2 and ubiquitination of uS3 in NGD is not manifested.

Although the mechanism of NGD has been studied using reporter genes, little is known about its endogenous targets. Ribosome footprinting is a powerful method for seeking cleavage sites of the endogenous targets (Guydosh et al., 2017). Recent sequencing-based strategy termed 5′ hydroxyl RNA sequencing (5′OH-seq) is a striking method to identify the 5′ ends of the cleaved intermediates (Peach et al., 2015; Ibrahim et al., 2018). Interestingly, NGD occurs under oxidative stress conditions (Simms et al., 2014), likely due to oxidized bases of mRNAs that interfere with base-pairing and cause aberrant translation elongation. The levels of K63-linked polyubiquitinated ribosomal proteins, translation factors, and various proteins were significantly increased upon oxidative stress by the down-regulation of deubiquitinating enzyme Ubp2 (Silva et al., 2015). These results strongly suggest a crucial role for K63-linked polyubiquitination of ribosomal proteins in NGD upon oxidative stress. To address the precise functions of K63-linked polyubiquitination in NGD, the essential E3 ubiquitin ligase and its target sites should be identified.

### A Disome as a Unit for Hel2-Dependent RQC and NGD in Yeast

**FIGURE 3:**
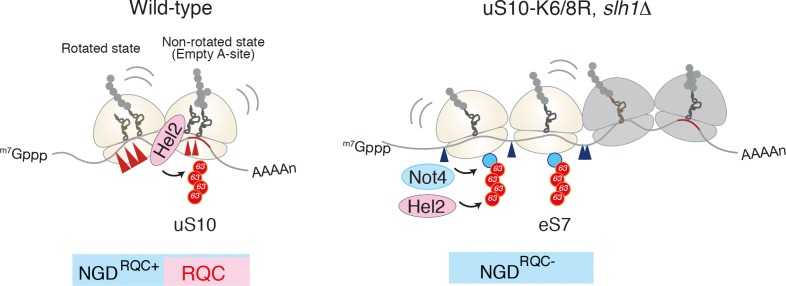
A unique structural interface to induce Hel2-driven quality control pathways. Model for quality control pathways induced by R(CGN)_12_-mediated translation arrest (Ikeuchi et al., 2019). Hel2-mediated ribosome ubiquitination is required both for canonical NGD (NGD^RQC+^) and RQC coupled to the disome, and that RQC-uncoupled NGD outside the disome (NGD^RQC-^) takes place in a Not4-mediated monoubiquitination dependent manner. The arrowheads indicate the endonucleolytic cleavages sites in NGD. The red line indicates the rare codon cluster. Left: the RQC pathway is intact, the leading ribosome that is stalled by the arrest sequence undergoes RQC. The uS10 ubiquitination and Slh1/Rqt2-dependent subunit dissociation induce the endonucleolytic cleavages in the disome. Right: In the absence of uS10 ubiquitination and Rqt2, RQC in the first ribosome, as well as NGD in the disome, are eliminated. RQC-uncoupled NGD^RQC-^ takes place upstream of the disome. The figure concept has been reproduced from the original article (Ikeuchi et al., 2019).

Recent study by Beckmann and Inada labs demonstrated that the mRNA cleavage by the CGA rare codon cluster is dependent on Hel2-mediated K63-linked polyubiquitination (Ikeuchi et al., 2019). The determination of the cleavage sites by primer extension revealed that endonucleolytic cleavage of an NGD reporter mRNA occurs at sites within a disome unit consisting of the stalled ribosome and the following colliding ribosome. This minimal ribosome collision unit is required to couple NGD and RQC via Hel2. The cleavages in a disome require the ubiquitination of uS10 at lysine 6th (K6) or 8th (K8) residues as well as the activity of the RQT component Slh1/Rqt2. Both Hel2-mediated ubiquitination of uS10 at K6 or K8 residues and Slh1/Rqt2 are essential for RQC, indicating that NGD and RQC are coupled via this ubiquitination event, and the NGD is referred to as the NGD^RQC+^ (Figure 3). The determination of the cleavage sites in the RQC-defective mutants in which Hel2-dependent ubiquitination is defective or Slh1/Rqt2 deletion mutant revealed that the NGD pathway could be dissected into two interdependent branches. In this alternative NGD pathway, endonucleolytic mRNA cleavages occur upstream of the stalled disome (referred to as the NGD^RQC-^; Figure 3). These cleavages require K63-linked polyubiquitination of ribosomal protein eS7. This polyubiquitination happens in a two-step mechanism, where the E3 ligase Not4 first monoubiquitinates eS7 which is followed by Hel2-mediated polyubiquitination. Finally, it was proposed that a dual role of Hel2 leading to two distinct NGD pathways, which require specific ubiquitination events on the stalled disome (Figure 3).

It is still unknown whether ribosome collision itself is essential for ribosome splitting and endoribonucleolytic cleavage, resulting in nascent peptide degradation and mRNA degradation. Hel2-mediated uS10 ubiquitination is required for RQC and NGD in a disome, and the disomes are preferred as targets for Hel2-mediated uS10 ubiquitination over monosomes. This raises the question of whether the endoribonucleolytic cleavage is required for RQC, which is an essential question to understand the mechanism of the coupled quality controls by ribosome stalling.

## Perspectives

Recent studies have clarified the novel molecular mechanisms how quality controls systems recognize stalling ribosome and eliminate aberrant products. However, many questions should be addressed including the relation between RQC and NGD, the roles of RQC factors in NGD. Future experiments and analyses will uncover the molecular mechanisms and biological functions of quality controls induced by ribosome stalling.

## Author Contributions

All authors listed have made a substantial, direct and intellectual contribution to the work, and approved it for publication.

## Conflict of Interest Statement

The authors declare that the research was conducted in the absence of any commercial or financial relationships that could be construed as a potential conflict of interest.
